# Supplementary material for: Detection and treatment of mental disorders in patients with coronary heart disease (MenDis-CHD): A cross-sectional study
Source: PLoS One. 2020 Dec 14;15(12):e0243800. doi: 10.1371/journal.pone.0243800 (PMC7735609; doi:10.1371/journal.pone.0243800)
Supplement: S1 Table — Abbreviations: SCID, Diagnostic and Statistical Manual of Mental Disorders. aNote: patients can have more than one SCID diagnosis. (DOCX) [file pone.0243800.s001.docx]

**Supporting Information Citation**

**S1 Table.**

|  | **Center** | | | **Total**  *N =* 364, *n* |
| --- | --- | --- | --- | --- |
|  | Hospitals  *N =* 107, *n* | Rehabilitation clinics  *N =* 157, *n* | Practices  *N =* 100, *n* |  |
| SCID-I Diagnoses ^a^ |  |  |  |  |
| No SCID diagnosis | 82 | 103 | 77 | 262 |
| Refused SCID testing | 5 | 5 | 2 | 12 |
| Positive SCID diagnoses | 26 | 61 | 29 | 102 |
| Positive SCID-I diagnoses ^a^  Alcohol dependence in remission (F10.21) | 3 | 3 | 3 | 9 |
| Cannabis abuse (F12.1) | - | 2 | - | 2 |
| Cocaine dependence, in remission (F14.21) | - | 1 | - | 1 |
| Multiple substance use disorder (F19.2) | - | - | 1 | 1 |
| Multiple substance induced psychotic disorder (F19.5) | - | 1 | - | 1 |
| Bipolar disorder, current episode depressed, moderate  (F31.32) | - | 1 | - | 1 |
| Bipolar disorder, most recent episode depressed, in partial  remission (F31.75) | 1 | - | - | 1 |
| Major depressive disorder, single episode, mild (F32.0) | 1 | 4 | - | 5 |
| Major depressive disorder, single episode, moderate (F32.1) | 5 | 7 | 5 | 17 |
| Major depressive disorder, single episode, in partial remission (F32.4) | 2 | 5 | 3 | 10 |
| Major depressive disorder, single episode, unspecified  (F32.9) | 1 | 1 | - | 2 |
| Major depressive disorder, recurrent, mild (F33.0) | - | 3 | - | 3 |
| Major depressive disorder, recurrent, moderate (F33.1) | 2 | 6 | 1 | 9 |
| Major depressive disorder, recurrent, in full remission  (F33.42) | 1 | - | - | 1 |
| Dysthymic disorder (F34.1) | - | - | 2 | 2 |
| Depressive disorder (self-declaration questionnaire) | 3 | 3 | 4 | 10 |
| Agoraphobia with panic disorder (F40.01) | - | 2 | 1 | 3 |
| Agoraphobia without panic disorder (F40.02) | - | - | 1 | 1 |
| Specific phobia (F40.2) | - | 2 | 1 | 3 |
| Panic disorder (F41.0) | 1 | - | - | 1 |
| Generalized anxiety disorder (F41.1) | 1 | - | - | 1 |
| Anxiety disorder, unspecified (F41.9) | 1 | 1 | 3 | 5 |
| Anxiety disorder (self-declaration questionnaire) | 3 | 6 | 3 | 12 |
| Post-traumatic stress disorder (F43.1) | - | 4 | - | 4 |
| Adjustment disorder (F43.2) | 1 | 9 | 1 | 11 |
