# Supplementary material for: Detection and treatment of mental disorders in patients with coronary heart disease (MenDis-CHD): A cross-sectional study
Source: PLoS One. 2020 Dec 14;15(12):e0243800. doi: 10.1371/journal.pone.0243800 (PMC7735609; doi:10.1371/journal.pone.0243800)
Supplement: S2 Table — Showing the difference in the current state of psychosomatic support between patients who had both a pre-existing MD diagnosis and were positively tested in our study (SCID-I) versus patients who had no pre-existing MD diagnosis but were positively tested (SCID-I). Abbreviations: MD, Mental Disorder; SCID, Diagnostic and Statistical Manual of Mental Disorders. aNote: All percentages relate to the maximum number of patients who were eligible for the presented questions. bNote: At least one cell was too small for the appropriate analysis. (DOCX) [file pone.0243800.s002.docx]

**Supporting Information Citation**

**S2 Table.**

|  | | **Pre-diagnosed MD diagnosis**  **AND positive SCID-I**  *N* ^a^ = 50, *n (%)* | | **No pre-diagnosed MD diagnosis**  **BUT positive SCID-I**  *N* ^a^ = 52, *n (%)* | | **P value** |
| --- | --- | --- | --- | --- | --- | --- |
| **Current state of psychosomatic support** | | | | | |  |
| Talking with physician about psychological problems | Yes  No | 39  11 | 78.0  22.0 | 23  27 | 46.0  54.0 | .001 |
| Actively approached by the physician on MD | Yes  No | 29  11 | 72.5  27.5 | 13  14 | 48.1  51.9 | .043 |
| **Diagnostics** | | | | | |  |
| Own perception of decline of mental well-being | Yes  No | 36  8 | 75.0  16.7 | 20  8 | 57.1  22.9 | .190 |
| Asked by others about decline of mental well-being | Yes  No | 20  26 | 41.7  54.2 | 12  19 | 36.4  57.6 | .216 |
| Psychological/psychiatric examination carried out | Yes  No | 40  8 | 83.3  16.7 | 6  24 | 19.4  77.4 | <.001 |
| Diagnosed positively MD symptoms | Yes  No | 31  12 | 64.6  25.0 | 1  26 | 3.2  83.9 | <.001 |
| Referral to diagnostics | Yes  No | 26  21 | 55.3  44.7 | 4  23 | 12.9  74.2 | <.001 |
| **Treatment** | | | | |  |  |
| Was a treatment for MD recommended | Yes  No | 31  13 | 67.4  28.3 | 6  19 | 23.1  73.1 | .001 |
| Currently undergoing psychotherapy | Yes  No | 20  25 | 43.5  54.3 | 2  23 | 7.7  88.5 | .007 |
| Currently undergoing medication therapy | Yes  No | 13  31 | 28.3  67.4 | -  23 | -  92.0 | .013 |
| **Access and barriers to health care** | | | | | |  |
| Sufficiently preserved information on the content and accessibility of psychological care | |  |  |  |  |  |
| Yes | | 22 | 47.8 | 8 | 32.0 | ^b^ |
| Only information about content of psychotherapy | | 4 | 8.7 | 1 | 4.0 |  |
| Only information on procuring a therapy | | 4 | 8.7 | 2 | 8.0 |  |
| No information | | 10 | 21.7 | 9 | 36.0 |  |
| Help with search for psychotherapeutic treatment | Yes  No | 22  14 | 48.9  31.1 | 5  10 | 20.0  40.0 | .101 |
