# Supplementary material for: Detection and treatment of mental disorders in patients with coronary heart disease (MenDis-CHD): A cross-sectional study
Source: PLoS One. 2020 Dec 14;15(12):e0243800. doi: 10.1371/journal.pone.0243800 (PMC7735609; doi:10.1371/journal.pone.0243800)
Supplement: S1 Appendix — Abbreviations: MD, mental disorders. (DOCX) [file pone.0243800.s003.docx]

**Supporting Information Citation**

**S1 Appendix. Additional analyses: Interaction between MD and healthcare.**

A two-way ANOVA with 'talking to the physician about MD' (Yes/No) and setting(clinic/rehabilitation/practice) as between-subjects factors revealed significant differences in HADS-scores on both between-subject factors. Patients who spoke to their physician about MD scored higher on HADS (*M*=10.8, *SD*=7.1) than patients who did not (*M*=8.7, *SD*=6.2, *F*(1,350)=5.79, *p*=.017, *eta²*=.016). The centers differed significantly on HADS-scores (*F*(2,350)=8.11, *p*<.001, *eta²*=.044). Patients in rehabilitation clinics scored noticeably higher on HADS than in the other two centers (*F*(2,350)=5.34, *p*=.005, *eta²*=.030; *M_reha_*=11.5, *SD_reha_*=6.9; *M_clinic_*=8.5, *SD_clinic_*=6.3; *M_practice_*=7.9, *SD_practice_*=5.9), being the leading cause for the interaction at hand.

However, an additional ANOVA on the group of patients that only 'talked once' to their physician about MD revealed only marginally significant differences on HADS scores between patients from clinics and rehabilitation clinics (*F*(5,146)=2.248, *p*=.053, *eta²*=.071).

Independent T-test revealed small (*t*(362)=3.140, *p*=.002, *d*=.366) but significant differences between MD and non-MD patients in how they rate their current health state. Non-MD patients gave a higher rating (*M_MD-_*=64.0, *SD_MD-_*=16.9) than MD patients (*M_MD+_*=57.7, *SD_MD+_*=17.9).

Pearson's *χ^2^* test examined differences in the number of MD and non-MD patients talking to their physicians about MD. The number of MD and non-MD patients differed across the categories of either talking or not talking to their physician (*χ^2^*(1)=15.798, *p*< .001). Adjust standardized residuals revealed that MD patients were overrepresented (i.e., observed more than expected) in the category 'talking to the physician' and were underrepresented in not talking to them, compared to non-MD patients.
