# Supplementary material for: Detection and treatment of mental disorders in patients with coronary heart disease (MenDis-CHD): A cross-sectional study
Source: PLoS One. 2020 Dec 14;15(12):e0243800. doi: 10.1371/journal.pone.0243800 (PMC7735609; doi:10.1371/journal.pone.0243800)
Supplement: Attachment — Submitted filename: Response to Reviewers.docx [file pone.0243800.s004.docx]

**Rebuttal letter with respond to each point raised by an academic editor and reviewers**

| **Journal requirements** | | **Author’s response** |
| --- | --- | --- |
| 1. Please ensure that your manuscript meets PLOS ONE's style requirements, including those for file naming. The PLOS ONE style templates can be found at  https://journals.plos.org/plosone/s/file?id=  wjVg/PLOSOne_formatting_sample_main_  body.pdf and  https://journals.plos.org/plosone/s/file?id=  ba62/PLOSOne_formatting_sample_title_  authors_affiliations.pdf | | We corrected the title page.  We placed the figure legend directly after the figure title on page 9.  We corrected the size of the headings in the result section from level 3 headings to level 2 headings. We made overall corrections in the whole text. |
| 2. We note that you have indicated that data from this study are available upon request. PLOS only allows data to be available upon request if there are legal or ethical restrictions on sharing data publicly. For information on unacceptable data access restrictions, please see http://journals.plos.org/plosone/s/data-availability#loc-unacceptable-data-access-restrictions. In your revised cover letter, please address the following prompts:  a) If there are ethical or legal restrictions on sharing a de-identified data set, please explain them in detail (e.g., data contain potentially identifying or sensitive patient information) and who has imposed them (e.g., an ethics committee). Please also provide contact information for a data access committee, ethics committee, or other institutional body to which data requests may be sent.  b) If there are no restrictions, please upload the minimal anonymized data set necessary to replicate your study findings as either Supporting Information files or to a stable, public repository and provide us with the relevant URLs, DOIs, or accession numbers. Please see http://www.bmj.com/ content/  340/bmj.c181.long for guidelines on how to de-identify and prepare clinical data for publication. For a list of acceptable repositories, please see http://journals.  plos.org/plosone/s/data-availability#loc-recommended-repositories.  We will update your Data Availability statement on your behalf to reflect the information you provide. | | We added an answer in the revised coverletter at the end of page 1 and at the beginning of page 2. |
| 3. One of the noted authors is a group, CoRe-Net study group. In addition to naming the author group and listing the individual authors and affiliations within this group in the acknowledgments section of your manuscript, please also indicate clearly a lead author for this group along with a contact email address. | | The lead author for the CoRe-Net Study group is Holger Pfaff. We added this information and the email address in the acknowledgements (p. 22, 521-522). |
| **Reviewer's responses to questions** | **Author’s response** | |
| 1. Is the manuscript technically sound, and do the data support the conclusions?  The manuscript must describe a technically sound piece of scientific research with data that supports the conclusions. Experiments must have been conducted rigorously, with appropriate controls, replication, and sample sizes. The conclusions must be drawn appropriately based on the data presented.  Reviewer #1: Yes  Reviewer #2: Partly | We adapted the manuscript following the recommendations of the reviewers and made the research questions and aims more specific. Further, the whole manuscript was revised and got a new structure. We added on several points more information (e.g. questionnaires in the ‘assessment’ section”). | |
| 2. Has the statistical analysis been performed appropriately and rigorously?  Reviewer #1: Yes  Reviewer #2: No | We adapted the manuscript following the recommendations of the reviewers, see especially the recommendation of reviewer #1 ‘3. Analysis’ and of reviewer #2: recommendation 6-14. We hope the statistical analysis is now performed appropriately and rigorously. | |
| 3. Have the authors made all data underlying the findings in their manuscript fully available?  The PLOS Data policy requires authors to make all data underlying the findings described in their manuscript fully available without restriction, with rare exception (please refer to the Data Availability Statement in the manuscript PDF file). The data should be provided as part of the manuscript or its supporting information or deposited to a public repository. For example, in addition to summary statistics, the data points behind means, medians and variance measures should be available. If there are restrictions on publicly sharing data—e.g. participant privacy or use of data from a third party—those must be specified.  Reviewer #1: Yes  Reviewer #2: Yes | No action required. | |
| 4. Is the manuscript presented in an intelligible fashion and written in standard English?  PLOS ONE does not copyedit accepted manuscripts, so the language in submitted articles must be clear, correct, and unambiguous. Any typographical or grammatical errors should be corrected at revision, so please note any specific errors here.  Reviewer #1: Yes  Reviewer #2: No | We made a language and grammar check for the whole manuscript. | |

| **Reviewer #1** | **Author’s response** |
| --- | --- |
| 1. In my opinion, because a range of different outcomes was analyzed, and some results were reported on the entire sample, people with mental disorders, or a comparison, the results and discussion could be restructured/revised to enhance the clarity of the manuscript. | We restructured both the results and the discussion as a consequence of your feedback. Both sections now are shaped in such a way that they address the aims of your study in the same sequence as they are described in the introduction (see page 3-5). To increase readability even more we decided to add different section headings (pages 9, 11, 13, 14, 15, 17, 18, 19) and focused content-wise on the differences between patients with and without mental disorders. |
| 2. Moreover, the manuscript could benefit from minor language editing. | We made a language and grammar check for the whole manuscript. |
| *Abstract  The use of bivariate statistics and rather descriptive nature of the study should be stated as early as in the abstract. | We add a statement in the abstract on p. 2, l. 40-41. |
| *Introduction  1. The authors state that “(...) evidence concerning the quality of psychosomatic care and trajectories within the healthcare system in CHD patients with MD is scarce.” (p. 4). However, there are some longitudinal studies available regarding trajectories of mental health (particularly anxiety and depression), quality of life, mental health treatment, and health care costs in patients with CHD, that are, in my opinion, not considered (e.g. DOI 10.1016/j.jpsychores.2017.10.015; 10.1159/000501502). In light of this, it should be stated more clearly what this study adds to the existing scientific literature.  On a different note, the introduction seems to be very short. Please add more details to better shape the state of current literature regarding mental disorders in patients with coronary heart disease. | Thank you for this point. We added the named publication and discussed the differences to our study on page 4, l. 104-107.  Furthermore, we added some information in the introduction, p. 3-5. |
| 2. The sentence „MenDis-CHD could successfully archive these aims.“ is, in my opinion, rather a conclusion and should be removed from the aim/introduction section. | We removed this sentence. |
| *Materials & Methods  1. For reasons of transparency, please elaborate on sampling technique. | We described the sampling technique in more detail in the “procedure” (p. 7/8, l. 190-194). |
| 2. In general, the description of the questionnaire was rather short and further details on the assessment of outcomes could be added. Please elaborate. | We added to every questionnaire more details under “assessments” on page 6 and 7. |
| 3. Analysis: Because the authors state that only a minority of the questionnaire items had subgroups that were to small for statistical analysis (p. 16), I am particularly interested why the analysis was on a bivariate level and there was no adjustment for confounding variables for the main outcomes, e.g. by means of regression. | Due to prior internal revisions, at some point it was decided to reduce possible type 1 errors by utilizing fewer tests. Therefore, the impact of methods for correcting for multiple tests like Bonferroni was minimized. The trade-off was lack of adjustment for confounding variables. We reduced the total number of tests and added correction for confounding variables to all analyses in tables 2 and 3, as well as the additional analyses. |
| *Strength & Limitations:  1. A major strength of this study, that is not mentioned in this section and could be added, is the thorough assessment of mental disorders by means of SCID-I. Having said this, as a positive HADS-screening was the prerequisite for the SCID assessment some MDs may also have been undetected. | Thank you for the good point. We added this strength with more details on page 20. Further, we added the second point as another limitation, also on page 20. |
| 2. Moreover, I feel that some statements in this section are a conclusion or result rather than a strength of the study and its design and I would recommend revising. | We revised this section and replaced the conclusions (page 20). |
| 3. In the limitations section I would strongly recommend to include the cross-sectional nature of the study and the assessment of health care use/sick days for the previous 12 months in self-report, which could be affected by recall bias. | Thank you for your recommendation, we added these points to the limitations on page 20. |
| *Tables  1. I was wondering why the results in the tables were presented by recruitment setting, as stratification by setting was not the aim/research question of the study and these results are not discussed in detail. | Indeed, after carefully re-reading the study it became apparent that we did not manage to properly convey the focus of our study. Thank you. As a result of your feedback, we restructured tables 1 and 2 and added a third (table 3), focusing more on the differences between MD and non-MD. See pages 10, 12 and 14. |
| 2. Percentages could be added in Supplementary Tables. | Tables and text have been split up to increase clarity. Descriptive measures such as percentages, as well as mean and SD values are now reported in the text, whereas statistical outcomes (p-values, confidence intervals, etc.) are presented in tables 2 and 3. Table 1 is an exemption, as this table also contains demographic information. |

| **Reviewer #2** | **Author’s response** |
| --- | --- |
| 1. There is no clear statement of the aims of the study in the abstract. The aim stated (to explore the state of healthcare recording diagnostics and treatment of MDs in secondary care in CHD patients with MD) is vague and cannot be tested. Three aims are stated in the Introduction, but again these are relatively vague and not clear (eg. aim 2: what ‘patients’ trajectories’ are you referring to? Clear aim needs to be articulated in both the abstract and the Introduction. | First, we replaced “aims” with “objective” in the abstract to state underline the exploratory nature of the study.  Second, we defined trajectories on p. 4, l. 97-104 and in the new formulated aims.  Third, we concretized the aims/hypotheses and added the new aims in the abstract (p. 2, l. 32-34). and in the introduction (p. 4, 104 - p.5. l. 118) |
| 2. There are sentences in the Introduction that do not make sense. For example, page 3 line 57-58: ‘…are both among the top ten disorders concerning year’s lives with disability worldwide’ and page 3 line 66 ‘the negative impact of MD on the incidence and prognosis…’ of what? Presumably this is on the CHD incidence and prognosis. The manuscript requires a thorough edit for grammatical correctness. | We corrected the sentences and added overall new information in the introduction, to make some points clearer.  Furthermore, we made a language and grammar check for the whole manuscript. |
| 3. Some of the crucial references are too old and therefore not relevant to today’s setting. For example, page 3 line 76: to support the point that ‘adherence to CHD guidelines in physicians seems to be generally low..’ a reference about acceptance of guidelines by family care physicians in 2002 is cited. It is highly likely that this citation does not reflect guideline acceptance at present. | Thank you for this, we replaced the publication from 2002 with a more recent one. |
| 4. Remove the term ‘suffered from’ in lines 108 and 111 on page 5 and line 185 on page 7. It is more appropriate to say ‘had angiographically documented CHD’ and ‘had severe and unstable physical or mental conditions’ and ‘had had myocardial infarction’. ‘Suffered’ is a subjective word, whereas ‘had’ is objective. | We removed the term “suffered from” in all three cases and replaced it with “had” to provide more objectiveness. |
| 5. In the Assessments section (page 5 lines 118-122), it is unclear whether the 158 item questionnaire was given to all patients or only to those who screened positive for MD. It seems that many of the items would not be relevant unless a person has a diagnosed MD. Indeed, the detail of the 158 item questionnaire is inadequate as the reader is unable to ascertain what is actually asked in this very extensive questionnaire. Has this questionnaire been used before? Who developed it? Has it been validated? Is it publicly available? All this information needs to be provided. | Thank you for the remark. In fact, we were wrong about the number of items. There was a total of 158 questions in the whole study (incl. HADS, EQ5D etc.). The questions in this section were 51, so we adjusted the number accordingly.  Further, we added all the details on p. 6 and 7 and gave example questions to each section. In addition, we added more information about the validation. |
| 6. In the Results section, on page 8 line 190-191, it is stated that “There were significant differences in trajectories of healthcare between CHD patients with and without MD”. Given that this is a key result of the study (relating to the aims), these results should be presented in a table. At present, the results are presented in sentences referring to days in hospital, days of sick leave etc, with mean number of days reported. However, there is no significance level reported for any of these statements so it is not possible for the reader to determine if these differences were statistically significant. Moreover, in some of these statements the n value is slotted into the sentence, which is not a conventional approach. As stated, this information should be presented comprehensively in a table. | Thank you for this important feedback. We changed table 2 and added table 3. Both tables now contain only statistical outcomes relevant for our aims, which should make text and tables more readable. Differences between MD and non-MD patients with regard to the trajectories now can be found in table 3 on page 14.  Additionally, all n values outside parenthesis were removed from the results section. |
| 7. Table 1 reports on differences based on patient recruitment setting (hospital, CR, cardiology practice). To my mind, these differences are largely methodological and should be stated in a simple sentence rather than in a table. For example, the sociodemographic differences are largely cofounded: CR participants are younger, more likely living alone and have fewer qualifications, whereas hospital and practice patients are older and more likely retired. By virtue of their younger age, it is not surprising then that the CR cohort have higher rates of anxiety, SCID diagnosis and unipolar depression – these trends are consistent with the literature that reports higher mental health problems in younger patients. Again, I reiterate, these are patterns that are a result of the recruitment strategies used in the study, rather than a key finding relevant to the aims, so they do not deserve to be put in Table 1 but instead should be commented on in describing the heterogeneity of the study sample. | This feedback is very important to us. The differences between settings are now merely mentioned in the additional analyses section (p. 15-16) of the results and in the discussion, and are no longer presented as main findings. As proposed, the impact of the recruitment strategy and its consequences are now commented on in the discussion.  In addition, confounding variables have been taken into account and all p-values of tables 2 and 3 were corrected. |
| 8. Instead of the current Table 1, a table comparing characteristics of MD vs. non-MD patients would be much more revealing to the reader. | Thank you, we implemented your suggestion (see table 1, p. 10) |
| 9. In the section titled Current state of diagnostics and treatment (page 10), there appear to be some errors. On line 233-234 it is stated that ‘Patients in rehabilitation clinics were most likely to report a decline in their mental wellbeing’, however according to Table 2 this difference was not significant (p=.639). Likewise, the statement on line 238-240 implies that these differences were significant (ie. rehab patients being informed about MD diagnosis, diagnosed more often, received an explanation and got treatment recommendations), whereas according to the data in Table 2 all these differences are not significant. Further, the statement that ‘4.1% had medication therapy’ (line 241) is again not consistent with the data presented in Table 2. | Outcomes from the section titled ‘Current state of diagnostics and treatment’ and outcomes from table 2 indeed did not always match, as some outcomes from the text came forth from analyses not mentioned in table 2. We increased consistency by not mixing outcomes related to center-differences and MD/non-MD differences anymore, as it makes it harder for the reader to follow. All discussed results now directly relate to the tables (pages 10, 12 and 14). |
| 10. Indeed, Table 2 is impossible to interpret. There is no indication throughout the table as to what is the total N for each variable, therefore the numbers (n) and percentages (%) do not correspond. For example, regarding ‘own perception of decline in mental wellbeing’ 20/107 is 18% not 58.8% as reported in the table. Without stating the total N for each variable, it is impossible to verify the data and therefore impossible for the reader to interpret the results. | We restructured our tables in a way that now, row percentages instead of column percentages are shown. This resolved the mentioned issue and should make it possible to interpret all data adequately. |
| 11. Again, the current Table 2 is not very informative to the reader and does not reflect the aims of the study. The study was not designed to compare the rates of MD and other variables between patients from each of the three study settings: as stated, these findings are merely an incidental bi-product of the methodology employed by the researchers. It would be much more informative to present a table of the differences in all these variables for patients with and without MD. | As requested, we changed table 2 so that it presents the differences in all variables for patients with and without MD. You can find table 2 on page 12. |
| 12. The patient satisfaction data would be better presented in a table, with all relevant questionnaire items shown, so that the reader can quickly scan the results. Currently, there is no actual data shown so it is impossible for the reader to interpret the satisfaction levels for each item, and the relative satisfaction between items. | All questions regarding patient satisfaction derived from the PACIC have been added to table 3 including all PACIC items on page 14. |
| 13. In the section on Interaction between MD and healthcare, why is the sentence reporting differences between rehab and other centres in HADS scores reported here? This belongs back with the earlier data on differences in patient characteristics across the three settings, not here in the section on MD and healthcare. | Originally this section was meant for additional analyses. In the light of the new structure of the results and the deletion of center-oriented analyses, we think that the analysis can remain in this section (which also has been renamed to ‘additional analyses’, see page 15). It is the only center-oriented analysis remaining. |
| 14. The final results reported on page 13-14 report on differences between MD ad Non-MD patients on talking and not talking to physicians. I cannot understand why this is of any clinical significance? It is completely self-evident that MD patients would talk with physicians about MD more often than would non-MD patients (talking about MD is clearly not relevant to someone who does not have MD). Can the authors explain the clinical relevance of this information, or exclude it? | Indeed, one would expect someone with MD to be more likely to seek aid regarding MD than someone who does not suffer from a mental disorder.  In our study, most participants were older than middle-aged people. Such a target group often is characterized by high sensitivity to (public) stigma regarding mental health treatment, see for example [10.1097/JGP.0b013e3181cc0366](https://dx.doi.org/10.1097%2FJGP.0b013e3181cc0366). Sensitivity to stigma could result in the use of coping strategies such as denial of the mental problems at hand. The analysis was carried out to exclude the possibility that our sample could be affected by said stigma, which could have severe consequences.  However, as these results are not our main findings and were not discussed in length, it causes no harm to exclude them. They were meant for information only. Please let us know if you would still advise to exclude the findings. |
| 15. In the Discussion, line 316 on page 14, it is stated that women were twice as likely to develop MDs. However, this has not been reported or mentioned in the Results (or I was unable to find it). Plus, why is this result refenced to citation number 26 if it is from the findings of the present study? | Thanks, we removed this sentence since it is not in scope of our (new) aims and wrote a new beginning of the discussion section to adapt it better to the aims, p. 16, l. 377-380. |
| 16. Overall, the Discussion needs to be presented in light of previous literature and studies regarding the detection and management of mental disorders in cardiac patients, and in the population more broadly. In its current form, it is not adequately nuanced, it does not adequately relate the study findings to the literature, it is not synthesised in a way which adds further illumination of the findings for the reader, and is largely a summary of some of the findings presented in the Results. | Thank you, we revised the whole discussion and made a new structure regarding the research aims in light of the literature of the introduction section. Further, we added new literature to discuss adequately the study findings. In addition, we eradicated the result summary from the discussion. |
