# Supplementary material for: Detection and treatment of mental disorders in patients with coronary heart disease (MenDis-CHD): A cross-sectional study
Source: PLoS One. 2020 Dec 14;15(12):e0243800. doi: 10.1371/journal.pone.0243800 (PMC7735609; doi:10.1371/journal.pone.0243800)
Supplement: Attachment — Submitted filename: Response to Reviewers.docx [file pone.0243800.s005.docx]

**Rebuttal letter with respond to each point raised by an academic editor and reviewers**

| **Journal requirements** | **Author’s response** |
| --- | --- |
| A rebuttal letter that responds to each point raised by the academic editor and reviewer(s). You should upload this letter as a separate file labeled 'Response to Reviewers'. | Done |
| A marked-up copy of your manuscript that highlights changes made to the original version. You should upload this as a separate file labeled 'Revised Manuscript with Track Changes'. | Done |
| An unmarked version of your revised paper without tracked changes. You should upload this as a separate file labeled 'Manuscript'. | Done |
| **Reviewer's responses to questions** | **Author’s response** |
| 1. If the authors have adequately addressed your comments raised in a previous round of review and you feel that this manuscript is now acceptable for publication, you may indicate that here to bypass the “Comments to the Author” section, enter your conflict of interest statement in the “Confidential to Editor” section, and submit your "Accept" recommendation.  Reviewer #1: (No Response)  Reviewer #2: All comments have been addressed | No action require­d |
| 2. Is the manuscript technically sound, and do the data support the conclusions?  The manuscript must describe a technically sound piece of scientific research with data that supports the conclusions. Experiments must have been conducted rigorously, with appropriate controls, replication, and sample sizes. The conclusions must be drawn appropriately based on the data presented.  Reviewer #1: Partly  Reviewer #2: Yes | We moved results unneccessary for our research aims (additional analyses) to the appendix and added a section in the data analysis section that describes which analyses were used to address the research aims. These measures should have made the manuscript more comprehensible. |
| 3. Has the statistical analysis been performed appropriately and rigorously?  Reviewer #1: Yes  Reviewer #2: (No Response) | No action required |
| 4. Have the authors made all data underlying the findings in their manuscript fully available?  The [PLOS Data policy](http://www.plosone.org/static/policies.action#sharing) requires authors to make all data underlying the findings described in their manuscript fully available without restriction, with rare exception (please refer to the Data Availability Statement in the manuscript PDF file). The data should be provided as part of the manuscript or its supporting information, or deposited to a public repository. For example, in addition to summary statistics, the data points behind means, medians and variance measures should be available. If there are restrictions on publicly sharing data—e.g. participant privacy or use of data from a third party—those must be specified.  Reviewer #1: Yes  Reviewer #2: Yes | No action required |
| 5. Is the manuscript presented in an intelligible fashion and written in standard English?  PLOS ONE does not copyedit accepted manuscripts, so the language in submitted articles must be clear, correct, and unambiguous. Any typographical or grammatical errors should be corrected at revision, so please note any specific errors here.  Reviewer #1: No  Reviewer #2: Yes | We made overall corrects in the whole text. |

| **Reviewer #1** | **Author’s response** |
| --- | --- |
| 1. General notes on wording 1.1. Please make sure to use abbreviations/wording uniformly to enhance readability: “EQ5D” vs. “EQ-5D”;“CHD patients” vs. “CHD-patients”; for mental disorders, sometimes “MD” is used for singular and plural forms, while at other times, “MDs” is used to indicate the plural form; “sick leave” vs. “days of illness”, etc. | Thank you for your revision, we made overall changes in the whole manuscript. |
| 1.2. “Trajectory”: In my opinion “trajectory” refers to the observation of individuals at several points in time which would permit the analysis of intra-individual change or trajectories over time using longitudinal datasets. Because this was a cross-sectional study with a retrospective assessment of preceding health care use, this should be made clearer, e.g. “previous health care use and days of sick leave within the last X months”. | We added your recommendation as a clearer definition of the specific use of the term ‘trajectories’ in our study on p. 4, l.99-101. |
| 1.3. “Diagnosis”: Particularly in the section “patients’ trajectories and detection rates” it is often not quite clear what the term “diagnosis” refers to - does it mean MD/SCID result in your study or a previously documented diagnosis, i.e. detection as a case by a previous physician? Please revise for clarity. | Thank you. To clarify this, we added information on p.1, l. 347-350, l. 355-356, l. 359-361. |
| 2. Introduction I originally suggested to state the relevance of the study in more detail as well as in light of some more recent longitudinal studies that were, in my opinion, not considered when considering health care use/ costs/quality of life associated with mental health problems and depression management in patients with CHD in the introduction (“originally p. 4, “(...) evidence concerning the quality of psychosomatic care and trajectories within the healthcare system in CHD patients with MD is scarce.”). While the authors stated to have included the references and discussed the difference to their study, I cannot find them in the revised manuscript. | We went back and realized that we included a different article than the ones you suggested. Now you can find the articles by Kuhlmann et al. and Palacios et al. in the introduction section, ll. 102-113. |
| 3. Methods Thank you for providing further information on the assessments, Unfortunately, even with the added details, it is still not clear to me which assessment and which statistical method was used for which reported result exactly. In line with this, by which assessment was the detection rate of mental disorders in healthcare assessed?  Further restructuring of the “Assessments”-Section by section headings could be helpful. Moreover, the exact method of analysis for each research question could be added to the Tables/briefly mentioned in the text; or each research question and method of analysis should be described in more detail in the “data analysis” section for reasons of transparency. | Thank you for your feedback. We added subsection headings in the assessment section to further underline the items that have been used to address the three research aims. A section about the trajectories and the detection rate was added in the assessment section (l. 180+). In addition, a new section was added at the end of the data analysis section (ll. 251-261), in which we break down the analyses used for each research question. |
| 4. Results 4.1. Please avoid the use of “marginally significant”. Because an alpha of 0.05 was set for all analyses as the threshold for significant effects, results are either significant at this threshold or they are not. In line with this, why are p-values over 0.05 (e.g., contact with psychotherapists: p = 0.085) printed in bold in Table 3? | We decided to exclude all results that did not reach the level of significance (p=.05). These results are not printed in bold anymore in table 3. |
| 4.2. Additional analyses on interaction: The consideration of the different recruitment settings was not described as an aim of the study, there seems to be no a priori hypothesis about this additional analysis, and the results are not discussed in the discussion section. In line with this and because the manuscript already includes many analyses and outcomes, I would suggest to fully exclude the additional analysis or put it completely into the appendix as an exploratory, additional analysis, with a brief reference to it in the main manuscript. | Indeed, there was no a priori hypothesis about the additional analyses. We moved the whole paragraph from the manuscript to a new appendix (S1 Appendix). |
| 5. Discussion 5.1. As far as I can tell, the study could not analyze whether treatment received for MDs was adequate according to treatment guidelines, such as the German S3-Leitlinien (i.e., details on type and dose of medication/detailed information on psychotherapy). However, treatment adequacy was discussed (l. 396), while the study analyzes to whether or not the physician talked about mental health with their patients? Please revise. | This is correct, our study could not analyze whether MD treatment was adequate according treatment guidelines. Therefore, we changed the paragraph and derive from our results from the ‘treatment’ section, p. 18, .424-426. |
| 5.2. In the strengths & limitations section it is described that participants who fulfilled the criteria for a SCID-diagnosis were offered immediate psychological support and further information on psychotherapeutic treatment. While this thoughtful offer seems to be part of the study design (and should therefore be mentioned in the methods section) it does not seem to immediately influence the results of this manuscript or their interpretation. Therefore, it should not be described as detailed in the strengths section. | We added this detail to our procedure in the methods section, p. 8, l., 201-204 and reduced the details in the strengths and limitations section p. 21, l. 509-510. |

| **Reviewer #2** | **Author’s response** |
| --- | --- |
| Reviewer #2: The authors have adequately addressed the reviewers' comments and the manuscript is now much improved. | Thank you every much for your approval. |
